# Supplementary material for: TYGS is an automated high-throughput platform for state-of-the-art genome-based taxonomy
Source: Nat Commun. 2019 May 16;10:2182. doi: 10.1038/s41467-019-10210-3 (PMC6522516; doi:10.1038/s41467-019-10210-3)
Supplement: Supplementary file 1 — Supplementary Information [file 41467_2019_10210_MOESM1_ESM.pdf]

Supplementary Information

Manuscript title: TYGS is an automated high-throughput platform for state-of-the-art genome-based taxonomy

Authors: Meier-Kolthoff et al.

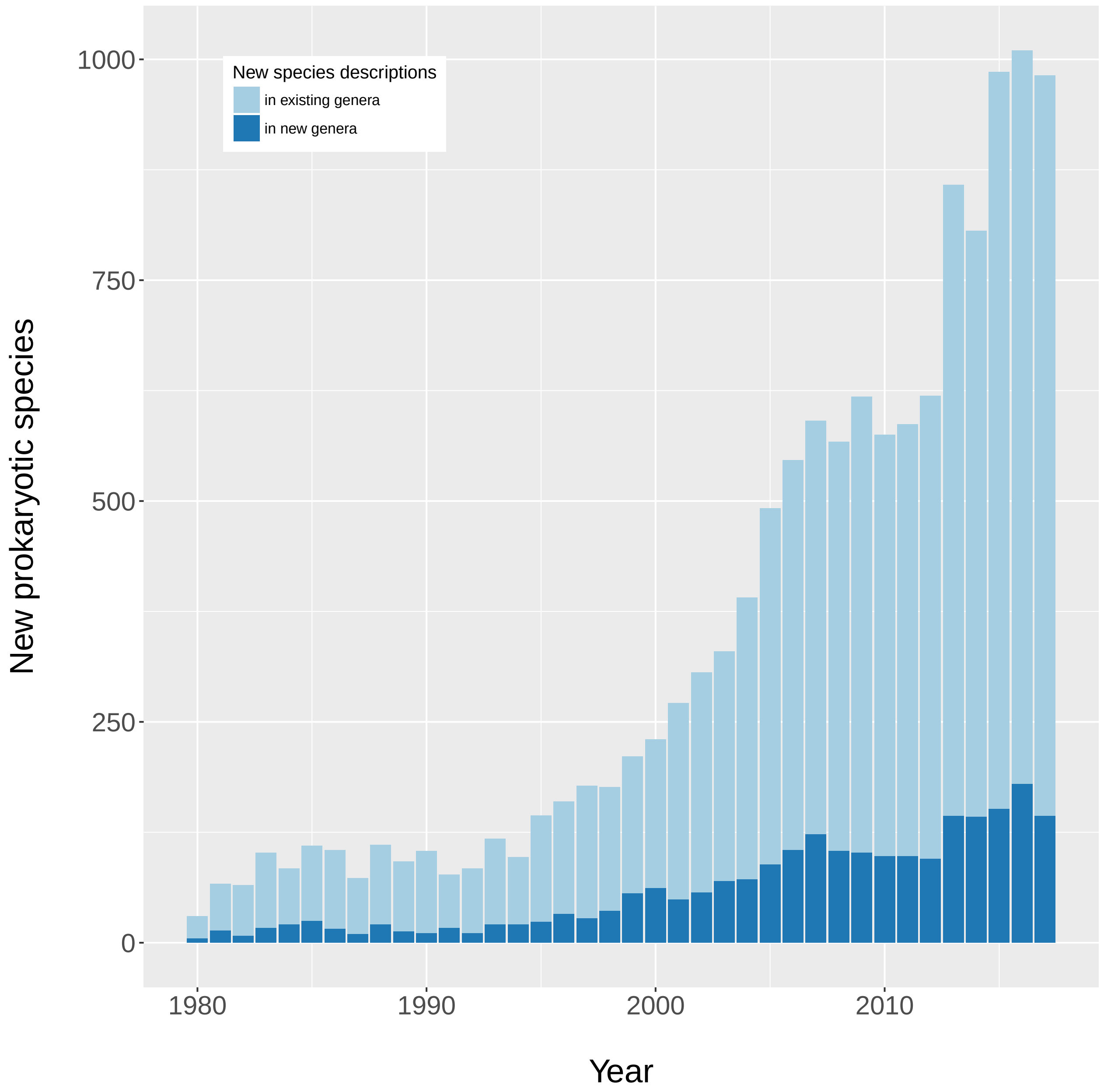

Supplementary Figure 1: New prokaryotic species per year from 1980 to 2017
New species are counted excluding both emendations and new combinations. The total number is further differentiated by the number of new descriptions in (i) new genera (dark blue) and (ii) existing genera (light blue). Data taken from DSMZ nomenclature database.

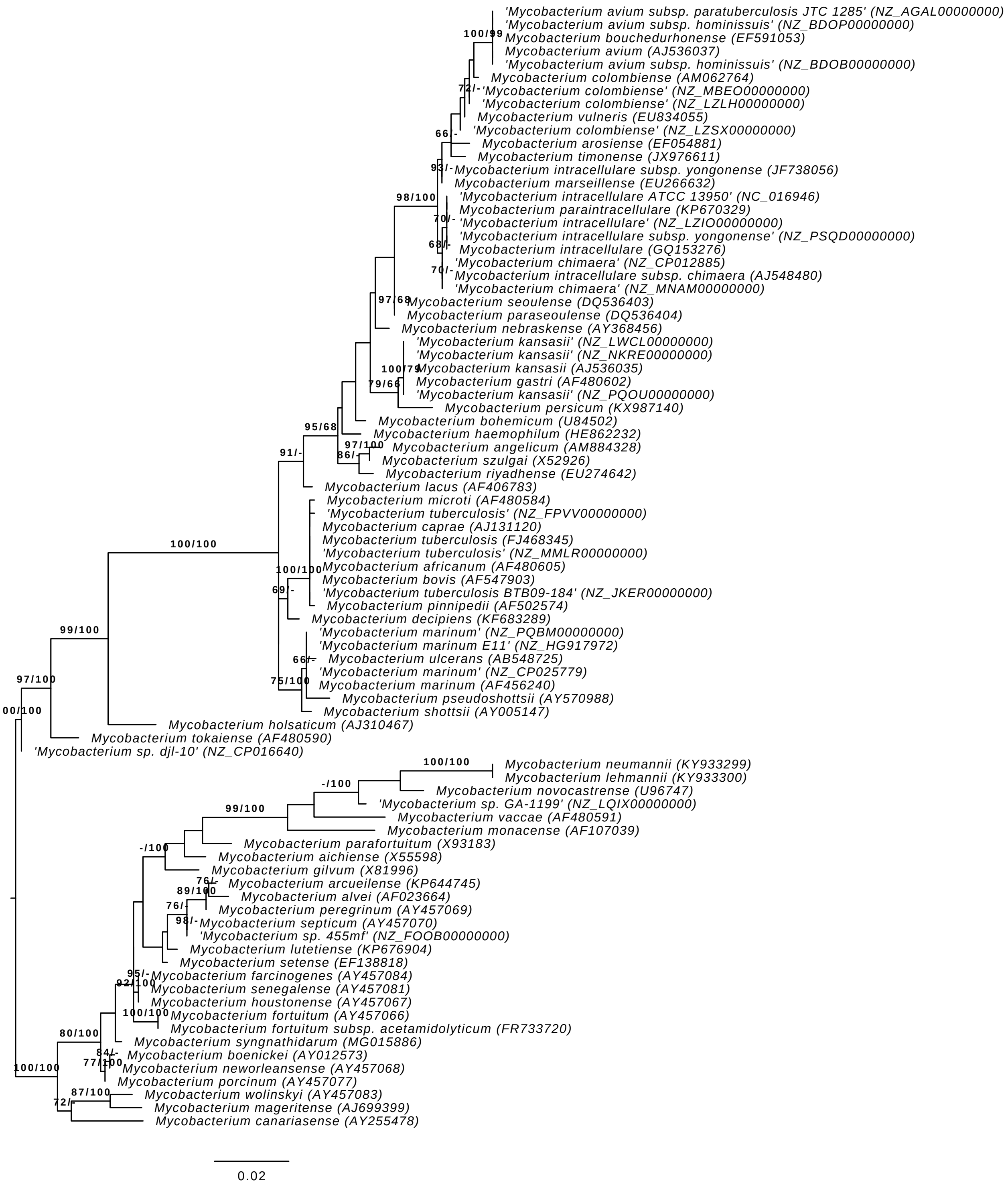

Supplementary Figure 2: ML tree of the *Mycobacterium* dataset inferred under the GTR+CAT model

The tree is rooted at the midpoint and branches are scaled in terms of the expected number of substitutions per site. The numbers above the branches are support values when larger than 60% from ML (left) and MP (right) bootstrapping. The tree was inferred through TYGS via the DSMZ gene phylogeny pipeline available under https://ggdc.dsmz.de/phylogeny-service.php.

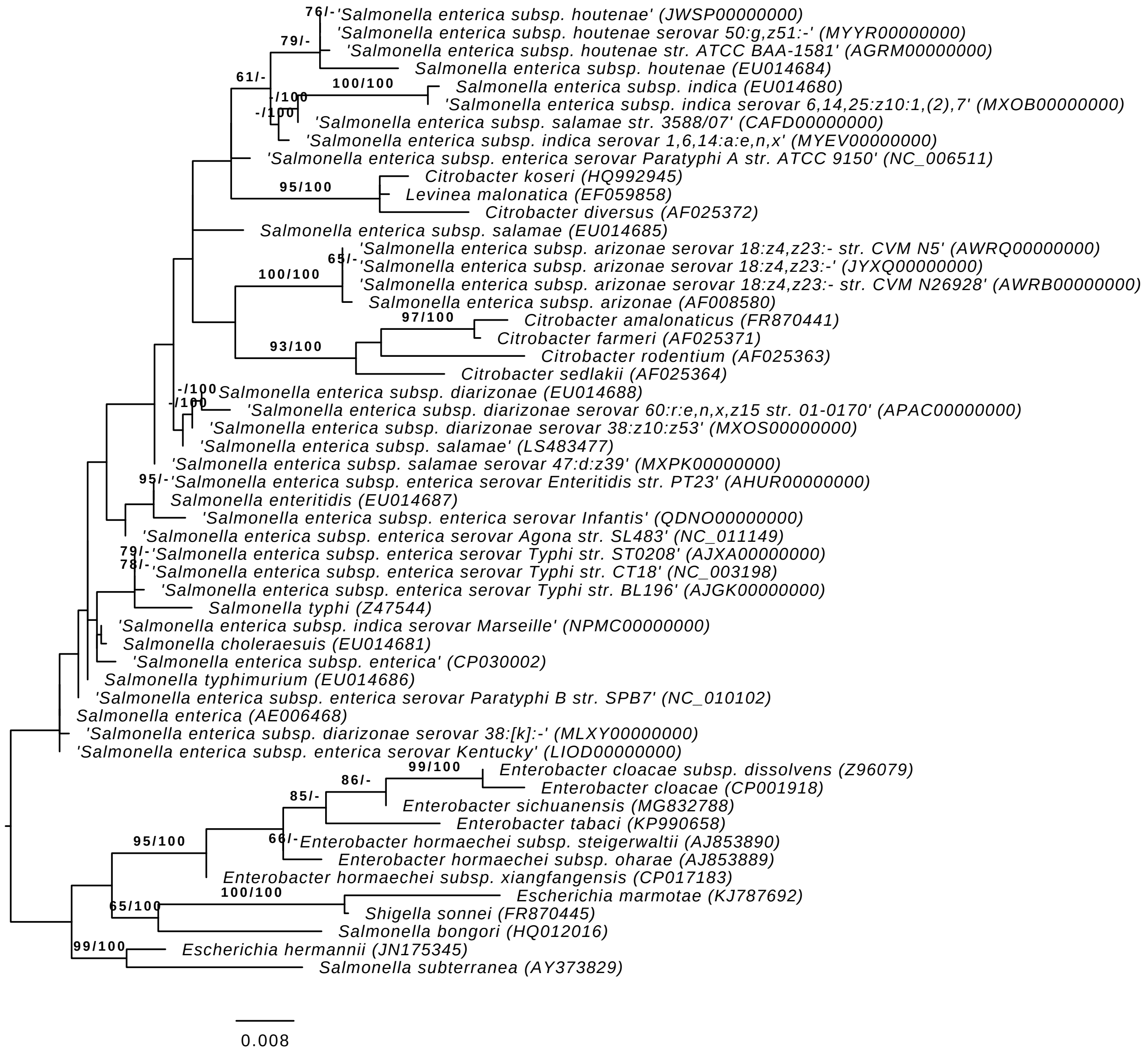

Supplementary Figure 3: ML tree of the *Salmonella* dataset inferred under the GTR+CAT model The tree is rooted at the midpoint and branches are scaled in terms of the expected number of substitutions per site. The numbers above the branches are support values when larger than 60% from ML (left) and MP (right) bootstrapping. The tree was inferred through TYGS via the DSMZ gene phylogeny pipeline available under https://ggdc.dsmz.de/phylogeny-service.php.

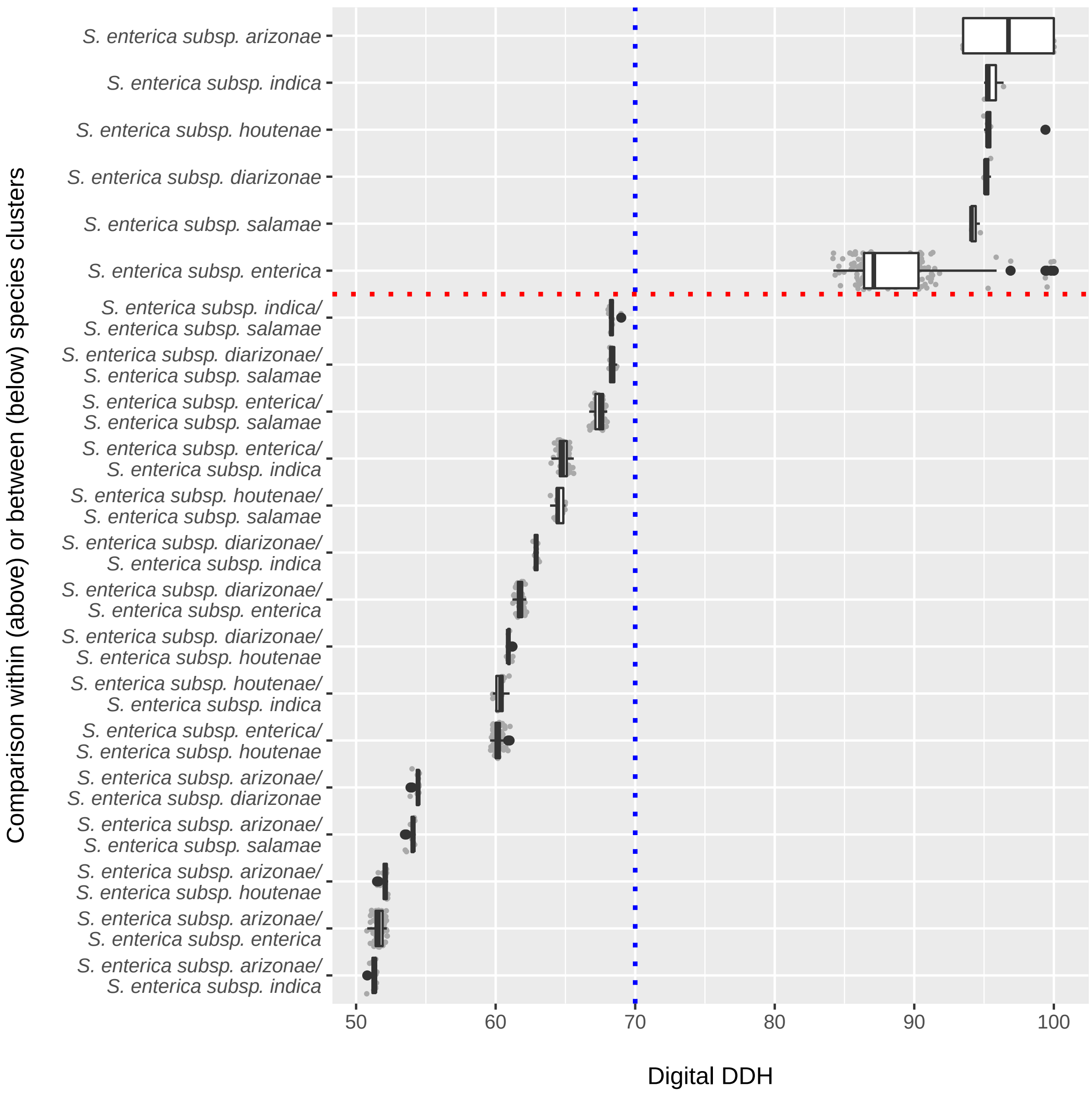

Supplementary Figure 4: Digital DDH distribution within and between *Salmonella enterica* species clusters

The intra-species cluster distribution (above dotted red line) comprises groups of strains with a high similarity throughout (dDDH values > 80 %), whereas the inter-species cluster distribution (below red line) reveals a clear separation between the hitherto subspecies (pairwise dDDH values below 70 % throughout). The dotted blue line indicates the 70 % DDH species delineation threshold. Error bars extending horizontally from the boxes (whiskers) indicate the variability outside the upper and lower quartiles. The underlying data is contained in Supplementary Table 3.
