# Supplementary material for: TYGS is an automated high-throughput platform for state-of-the-art genome-based taxonomy
Source: Nat Commun. 2019 May 16;10:2182. doi: 10.1038/s41467-019-10210-3 (PMC6522516; doi:10.1038/s41467-019-10210-3)
Supplement: Supplementary file 3 — Reporting Summary [file 41467_2019_10210_MOESM3_ESM.pdf]

nature research

Corresponding author(s): *Double-blind peer review submissions: write DBPR and your manuscript number here instead of author names.*

Reporting Summary

Nature Research wishes to improve the reproducibility of the work that we publish. This form provides structure for consistency and transparency in reporting. For further information on Nature Research policies, see Authors & Referees and the Editorial Policy Checklist.

Statistical parameters

When statistical analyses are reported, confirm that the following items are present in the relevant location (e.g. figure legend, table legend, main text, or Methods section).

n/a	Confirmed	
☒	☐	The exact sample size (n) for each experimental group/condition, given as a discrete number and unit of measurement
☒	☐	An indication of whether measurements were taken from distinct samples or whether the same sample was measured repeatedly
☒	☐	The statistical test(s) used AND whether they are one- or two-sided<br>*Only common tests should be described solely by name; describe more complex techniques in the Methods section.*
☒	☐	A description of all covariates tested
☒	☐	A description of any assumptions or corrections, such as tests of normality and adjustment for multiple comparisons
☒	☐	A full description of the statistics including central tendency (e.g. means) or other basic estimates (e.g. regression coefficient) AND variation (e.g. standard deviation) or associated estimates of uncertainty (e.g. confidence intervals)
☒	☐	For null hypothesis testing, the test statistic (e.g. F, t, r) with confidence intervals, effect sizes, degrees of freedom and P value noted<br>*Give P values as exact values whenever suitable.*
☒	☐	For Bayesian analysis, information on the choice of priors and Markov chain Monte Carlo settings
☒	☐	For hierarchical and complex designs, identification of the appropriate level for tests and full reporting of outcomes
☒	☐	Estimates of effect sizes (e.g. Cohen's d, Pearson's r), indicating how they were calculated
☒	☐	Clearly defined error bars<br>*State explicitly what error bars represent (e.g. SD, SE, CI)*

Our web collection on statistics for biologists may be useful.

Software and code

Policy information about availability of computer code

Data collection

TYGS fulfils end-user requirement of the journal for web tools by providing the web server free and publicly accessible with any modern web browser (http://blogs.nature.com/methagora/2014/02/guidelines-for-algorithms-and-software-in-nature-methods.html).

The TYGS database is implemented in the object-relational database system PostgreSQL (https://www.postgresql.org/). The TYGS workflow engine is written in the programming language Ruby (https://www.ruby-lang.org/en/), whereas the TYGS web server is implemented in Rails 5, a modern web application development framework (https://rubyonrails.org/). The web application makes use of Bootstrap 3.3.7, a free and open-source front-end framework (library) for designing websites and web applications (https://getbootstrap.com/), and PhyD3 for the visualization of phylogenetic trees.

Data analysis

TYGS fulfils end-user requirement of the journal for web tools by providing the web server free and publicly accessible with any modern web browser (http://blogs.nature.com/methagora/2014/02/guidelines-for-algorithms-and-software-in-nature-methods.html).

The TYGS database is implemented in the object-relational database system PostgreSQL (https://www.postgresql.org/). The TYGS workflow engine is written in the programming language Ruby (https://www.ruby-lang.org/en/), whereas the TYGS web server is implemented in Rails 5, a modern web application development framework (https://rubyonrails.org/). The web application makes use of Bootstrap 3.3.7, a free and open-source front-end framework (library) for designing websites and web applications (https://getbootstrap.com/), and PhyD368 for the visualization phylogenetic trees.

For manuscripts utilizing custom algorithms or software that are central to the research but not yet described in published literature, software must be made available to editors/reviewers upon request. We strongly encourage code deposition in a community repository (e.g. GitHub). See the Nature Research guidelines for submitting code & software for further information.

Data

Policy information about availability of data

All manuscripts must include a data availability statement. This statement should provide the following information, where applicable:

- Accession codes, unique identifiers, or web links for publicly available datasets
- A list of figures that have associated raw data
- A description of any restrictions on data availability

All exemplary sequence data used in this study are publicly available via the specified GenBank accessions.

Field-specific reporting

Please select the best fit for your research. If you are not sure, read the appropriate sections before making your selection.

☒ Life sciences ☐ Behavioural & social sciences ☐ Ecological, evolutionary & environmental sciences

For a reference copy of the document with all sections, see nature.com/authors/policies/ReportingSummary-flat.pdf

Life sciences study design

All studies must disclose on these points even when the disclosure is negative.

Sample size	not applicable
Data exclusions	not applicable
Replication	not applicable
Randomization	not applicable
Blinding	not applicable

Reporting for specific materials, systems and methods

Materials & experimental systems

n/a	Involved in the study	
☒	☐	Unique biological materials
☒	☐	Antibodies
☒	☐	Eukaryotic cell lines
☒	☐	Palaeontology
☒	☐	Animals and other organisms
☒	☐	Human research participants

Methods

n/a	Involved in the study	
☒	☐	ChIP-seq
☒	☐	Flow cytometry
☒	☐	MRI-based neuroimaging
