# Supplementary material for: TYGS is an automated high-throughput platform for state-of-the-art genome-based taxonomy
Source: Nat Commun. 2019 May 16;10:2182. doi: 10.1038/s41467-019-10210-3 (PMC6522516; doi:10.1038/s41467-019-10210-3)
Supplement: Supplementary file 4 — Description of Additional Supplementary Files [file 41467_2019_10210_MOESM4_ESM.pdf]

Description of Additional Supplementary Files

File Name: Supplementary Data 1
Description: This table contains the 16S rRNA gene sequence similarities among the type strains of the analysed *Mycobacterium* dataset.

File Name: Supplementary Data 2
Description: This table contains the 16S rRNA gene sequence similarities among the type strains of the analysed *Salmonella* dataset.

File Name: Supplementary Data 3
Description: This table contains the data used for the generation of Supplementary Figure 4.
